# Prevalence of respiratory epithelial adenomatoid hamartomas (REAH) associated with nasal polyposis: an epidemiological study – how to diagnose

**DOI:** 10.1016/j.bjorl.2021.09.009

**Published:** 2021-11-10

**Authors:** Maria Julia Abrão Issa, Vitor Ramos Ribeiro de Oliveira, Flavio Barbosa Nunes, Luís Otávio Giovanardi Vasconcelos, Luiz Felipe Bartolomeu Souza, Giancarlo Bonotto Cherobin, Roberto Eustáquio Santos Guimarães

**Affiliations:** a Universidade Federal de Minas Gerais (UFMG), Programa de Otorrinolaringologia, Belo Horizonte, MG, Brazil; b Universidade Federal de Minas Gerais (UFMG), Belo Horizonte, MG, Brazil; c Universidade Federal de Minas Gerais (UFMG), Faculdade de Medicina, Departamento de Otorrinolaringologia, Belo Horizonte, MG, Brazil; d Núcleo de Otorrino BH, Belo Horizonte, MG, Brazil; e Universidade Federal de Minas Gerais (UFMG), Hospital Universitário, Belo Horizonte, MG, Brazil; f Universidade de São Paulo, Ribeirão Preto, SP, Brazil; g Academia de Medicina de Minas Gerais, Belo Horizonte, MG, Brazil

**Keywords:** REAH, respiratory epithelial adenomatoid hamartoma, Nasal polyposis, Olfactory cleft, Nasal computed tomography scan

## Abstract

**Objectives:**

To highlight the prevalence of respiratory epithelial adenomatoid hamartomas in the olfactory cleft of patients with nasal polyposis. To demonstrate characteristics indicative of hamartoma on the CT scans of paranasal sinuses during surgery and in histopathological exams.

**Methods:**

Cross-sectional study carried out in Hospital das Clínicas da UFMG and Núcleo de Otorrino BH. We performed 114 nasal endoscopic surgeries for polyposis, between February 2015 and November 2019. We assessed the olfactory cleft width in all preoperative CT scans. Upon seeing an indication of hamartoma on the CT scan, we took a tissue sample from the olfactory cleft during the surgery and sent for histopathological exam. We referred the samples to a pathologist experienced in the anatomopathological diagnosis of respiratory epithelial adenomatoid hamartomas.

**Results:**

Of the 114 patients with polyposis, 54 (47.4%) had olfactory cleft enlargement and, 100% of them had tissue with a dense and hardened polypoid aspect, with a slight cerebriform appearance in this region during the surgery. Histology confirmed a respiratory epithelial adenomatoid hamartoma.

**Conclusion:**

This observation suggests that the presence of hamartomas in polyposis is common, but underdiagnosed.

**Level of evidence:**

Step 3 (Level 3).

## Highlights

• Olfactory cleft enlargement and opacification suggests hamartoma.
• Individualized anatomopathological exam of material from the olfactory cleft.
• Material examined by experienced pathologist.
• Almost half of the patients with Nasal Polyps have hamartomas.

## Introduction

Albretch introduced the term hamartoma (from the ancient Greek *hamartia*, meaning error, and *-oma*, benign growth) in 1904.^1^ Hamartomas are common in the liver, spleen, lung, kidney, and intestine, but they may also occur in the upper respiratory tract.

The Respiratory Epithelial Adenomatoid Hamartomas (REAH) is a subtype of hamartoma. Wenig and Heffner coined this term in 1995, to describe a benign intranasal glandular proliferation covered by multilayer ciliated respiratory epithelium, usually with mixed mucocytes.^2^ It may be present alone in the nasal cavity or in association with Nasal Polyps (NP). Usually found in the olfactory cleft, they have radiological and histopathological characteristics that enable us to differentiate them from other nasal polyps. Patients with REAH have symptoms similar to those of patients with inflammatory nasosinusal diseases, and olfactory complaints are common.

Although previously described in the literature as a rare entity, Nguyen et al.^3^ have already demonstrated a high prevalence of REAH associated with nasal polyps in their series.

This study focuses on the epidemiology and diagnosis of REAH associated with nasal polyps. We will not address hamartomas alone in the olfactory cleft.

## Objectives

To alert physicians on the prevalence of Respiratory Epithelial Adenomatoid Hamartomas (REAH) in the Olfactory Cleft (OC) of patients with Nasal Polyps (NP); and demonstrate characteristics indicative of REAH on the Computed Tomography Scan of the paranasal sinuses (CT), during surgery and in histopathological exams.

## Methods

The Ethics Committee – CAAE, approved this study: 07387312.5.0000, being a cross-sectional study carried out between January 2015 and November 2019, in two otorhinolaryngological reference centers of Belo Horizonte-MG, Hospital das Clínicas da UFMG and Núcleo de Otorrino BH. We selected patients with surgical indication for the treatment of Chronic Rhinosinusitis with Nasal Polyps (CRSwNP) during the study period.

**Figure 1:**
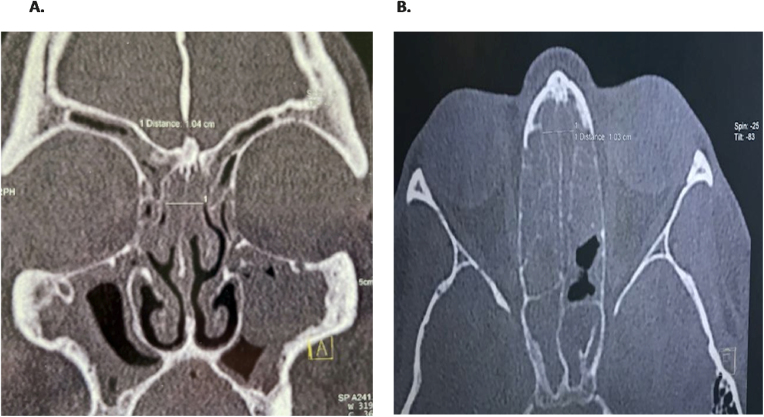
Respiratory Epithelial Aadenomatoid Hamartoma (REAH) associated with chronic rhinosinusitis, with an enlarged and opaque olfactory cleft (A, coronal section; B, axial section of a different patient).

**Figure 2:**
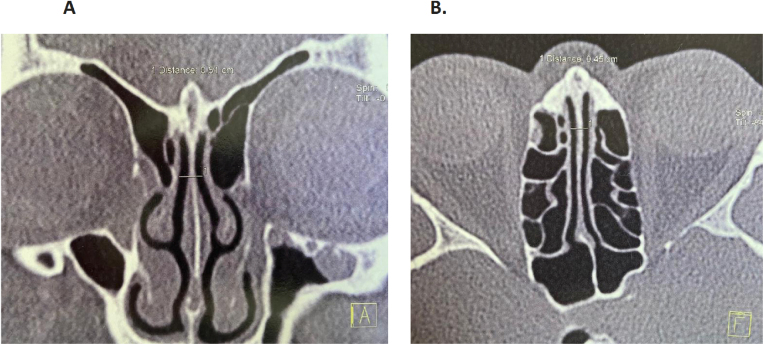
Normal length of the olfactory cleft (A, coronal section; B, axial section).

All the patients underwent preoperative CT analysis of the Olfactory Cleft (OC) to diagnose suspected cases of REAH. Olfactory clefts with a width greater than or equal to 10 mm between the right and left lateral limits were considered enlarged. Enlarged and opacified OC were considered potentially affected by REAH.^4^, ^5^ We excluded patients with CRS without Nasal Polyps (CRSWoutNP), those with concomitant neoplasms or those having hamartoma alone without chronic inflammatory nasosinusal disease (Figure 1, Figure 2).

**Figure 3:**
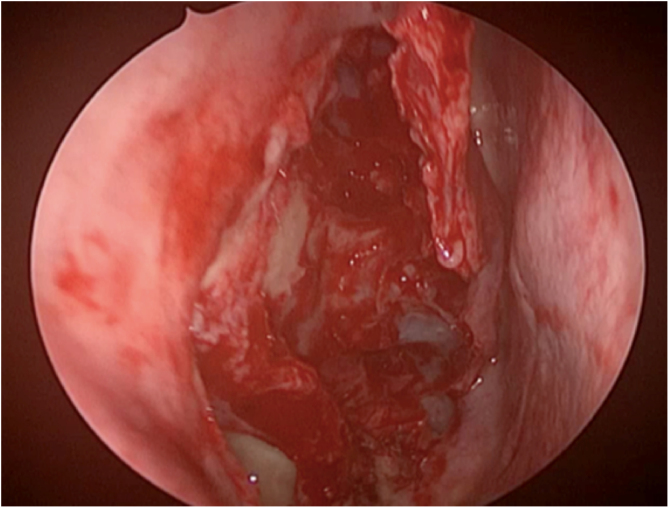
Proper anatomical aspect of the right nasal cavity after nasalization, removal of lesions, removal of hamartomas and olfactory cleft decompression.

All these patients were operated under the supervision of the same surgeons, and we performed endonasal surgery with the nasalization technique, following R Jankowski’s principles.^6^, ^7^ During the surgical procedure, the OC were evaluated. In those patients who had a lesion in this region, we tried to remove it completely, sometimes even exposing threads of the olfactory nerve through transparency. We sent the material removed from the OC for anatomopathological analysis in a flask different from those of other polyps. We referred this material to a pathologist familiar with the histological aspects of REAH (Fig. 3).

We stratified the patients by gender, and we calculated their mean age, and we separated them into 2 groups: NP + REAH; NP without REAH (Table 1).

**Table 1:** Epidemiological distribution by gender and age in the groups Nasal Polyps (NP) + Respiratory Epithelial Adenomatoid Hamartomas (REAH) and NP without REAH.

Distribution by gender and age of patients			
	Overall	NP without REAH	NP + REAH
Male	70 (61.4%)	36 (60%)	34 (63%)
Female	44 (38.6%)	24 (40%)	20 (37%)
Mean age	49 anos	48 anos	51 anos

## Results

**Figure 4:**
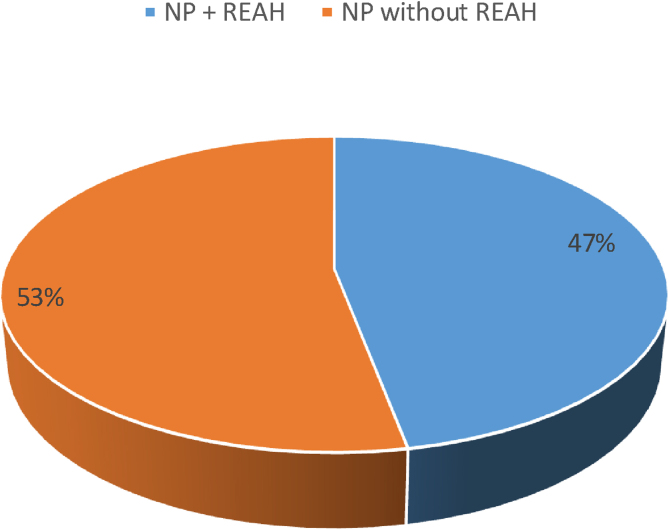
Prevalence in percentage of Respiratory Epithelial Adenomatoid Hamartomas (REAH) + Nasal Polyps (NP) – blue area; and NP without REAH – orange area.

**Figure 5:**
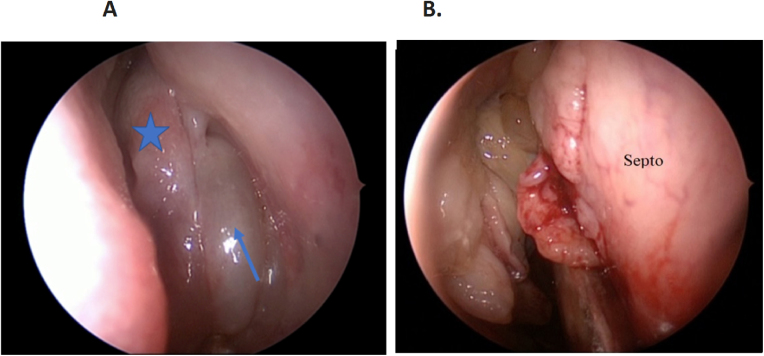
(A) Left-side middle meatus nasal polyp (blue arrow) and olfactory cleft hamartoma (blue star). (B) Extensive hamartoma in the right-side olfactory cleft, with septal invasion.

**Figure 6:**
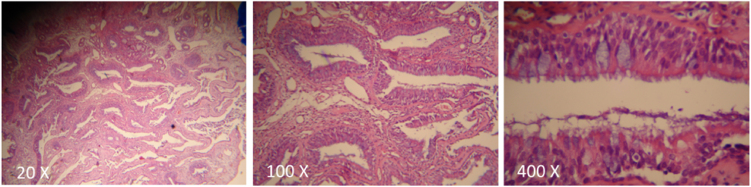
Histopathological findings of a Respiratory Epithelial Adenomatoid Hamartoma (REAH), 20×, 100× and 400× magnifications.

Of the 114 patients with polyposis, 54 (47.4%) had an enlargement of the OC (Fig. 4). In 100% of the patients with OC widening, the tissue with a polypoid mass-like appearance was denser and more indurated than the nasosinusal polyps,^8^ with a slight cerebriform, pink or sometimes yellowish appearance in this region during the surgery (Fig. 5), and the tissue’s anatomopathological study confirmed that it was REAH, through the observation of a typical histopathological aspect (Fig. 6).

As for gender, 70 patients were male (61.4%) and 44 female (38.6%), with an overall mean age of 49 years. In the NP group without REAH, the mean age was 48 years; 36 (60%) patients were males and 24 (40%) were females. In the NP group of + REAH, we had a mean age of 51 years, 34 (63%) males and 20 (37%) females (Table 1).

## Discussion

It was only in 2005 that REAH was added to the World Health Organization's classification of tumors. It is a relatively unknown entity among radiologists, otolaryngologists and pathologists; thus, underdiagnosed. Respiratory Epithelial Adenomatoid Hamartomas (REAH) of the nose have always been described as a rare entity and were limited to case reports up to a few years ago, being usually reported in retrospective studies involving the histopathological results of the set of polyp material. There are four distinct histopathological types of hamartomas described in the nasosinusal tract: congenital hamartoma, seromucinous hamartoma, mesenchymal hamartoma, and Respiratory Epithelial Adenomatoid Hamartoma (REAH). In this paper, we focus only on REAH from the OC of patients with Nasal Polyps (NP). According to Jankowski et al.,^3^, ^4^ hamartomas are usually found in the fifth decade of life, with a male to female ratio of 1.5:1 and occurrence of REAH in 40.57% of patients with polyposis. Findings which are similar from ours.

Patients with REAH have symptoms similar to those of patients with inflammatory nasosinusal diseases such as nasal obstruction, nasal discharge, facial pain, facial pressure, headache, epistaxis or ear fullness. Upon anterior rhinoscopy and nasal endoscopy, REAH appear as polypoid masses that are denser and more indurated than nasosinusal polyps,^9^ with a slight cerebriform, pinkish or sometimes yellowish appearance.^10^ They can have different sizes, being uni or bilateral, and are found in the olfactory cleft, between the septum and the middle concha. They are often covered by polyps that extend from the middle or superior meatus to the rest of the nasal cavity, which makes it difficult to diagnose them by nasal endoscopy. The lesion can be seen in two ways: isolated in the nasal cavity or associated with chronic inflammatory processes such as in NP, this being its most prevalent form and, in most cases it is bilateral.^3^, ^4^, ^9^, ^11^ The main differential diagnosis is inflammatory polyp, but there are other possibilities such as inverted papillomas, nasal adenocarcinoma, encephalocele and estesioneuroblatoma.^4^, ^12^, ^13^, ^14^ It is common for these patients to complain of their smell, and this symptom may or may not be associated with altered taste.

The etiology and pathophysiology of REAH are not precisely known, and the most acceptable hypothesis is that REAH is a hyperplastic disease, highly associated with the non-olfactory epithelium that covers the OC. This hyperplasia can be induced by the chronic inflammatory process associated with the development of nasal polyps in the ethmoidal labyrinth.^3^

The possibility of diagnosing REAH in patients with CRSwNP is directly related to the care in routinely looking at the OC in Computerized Tomography of the Facial Sinuses (FS CT) in coronal and axial views. Radiological changes may be present throughout the length of the OC or be located more anteriorly, or more posteriorly. In the presence of opacification and/or enlargement of the OC alone, or in the occurrence of NP, respiratory hamartoma should be suspected.^15^, ^16^ Tomographic widening greater than or equal to 10 mm, without bone erosion, are the main suspicious parameters.^4^, ^5^, ^17^ These findings indicate that we should make a detailed surgical exploration of this region in search of the lesion (Figure 1, Figure 2). In the present study, all 54 patients diagnosed with REAH associated with NP, had opacification and enlargement equal to or greater than 10 mm of the OC.

The definitive treatment for REAH is complete resection of the lesion, which rarely recurs after surgery, with no reports of malignant transformation^18^, ^19^, ^20^ (Fig. 3).

For the correct diagnosis of REAH, it is necessary to collect the sample from the OC region, as close as possible to the cribriform plate, and sent it for histopathological examination in an individual bottle, separately from the material collected from other regions (Fig. 5). The histological aspect is characterized by the presence of glandular proliferation with a polypoid aspect, which tends to be submucosal.^21^, ^22^ The glands are lined with ciliated respiratory epithelium originating from the superficial respiratory epithelium, and typically round to oval in shape, small to medium in size, with prominent dilation.^21^, ^23^ Stromal tissue separates the glands. Glandular complex growth and cribriform architecture are absent. The epithelium may be cuboidal or flat, there is frequent and mucinous gland metaplasia. Occasionally, the glandular lumen is filled with amorphous mucinous or eosinophilic material. There can be stromal hyalinization, but it is not present in all cases. There are no reports of dysplastic and neoplastic changes, confirming the benign nature of the lesion. Long-term chronic inflammation and the formation of polyps in the respiratory epithelium are considered the etiological precursors of REAH, which helps to explain chronic sinusitis as a symptom present in this process^21^ (Fig. 6). The main histopathological differential diagnosis of REAH is the inflammatory polyp – due to the overlapping of clinical, morphological, therapeutic and biological characteristics. Both lesions may show fibroblastic and vascular proliferation, stromal edema, mixed inflammatory cell infiltrate, and seromucinous gland proliferation. However, inflammatory polyps do not have overt adenomatoid proliferation and stromal hyalinization, which, when present, favor the diagnosis of REAH. The pathologist's familiarity with this diagnosis is crucial. We recommend a review of the histopathological examination in patients with evident clinical suspicion and negative histopathological diagnosis of REAH.

## Conclusion

REAH in the olfactory cleft should not be considered an uncommon finding^24^. One should actively look for this diagnosis in all patients with NP. In our series, we noticed a high prevalence of REAH associated with NP, corresponding to 47.4% of all patients in the study.

## Conflicts of interest

The authors declare no conflicts of interest.
